# The effects of exercise based on adherence to ACSM recommendations on pulmonary function and quality of life in adults with asthma: a systematic review and meta-analysis

**DOI:** 10.3389/fphys.2025.1548382

**Published:** 2025-05-15

**Authors:** Jinhan Li, Jianxu Zhao, Jiahao Wei, Baofa Wu, Wuzhuang Sun

**Affiliations:** ^1^ Department of Respiratory and Critical Care Medicine, The First Hospital of Hebei Medical University, Shijiazhuang, China; ^2^ Department of Neurobiology, Hebei Medical University, Shijiazhuang, China

**Keywords:** bronchial asthma, health related quality of life, exercise intervention, ACSM exercise recommendations, respiratory function

## Abstract

**Background:**

Adherence to ACSM exercise guidelines is linked to improved clinical outcomes in asthma patients, yet its effects on pulmonary function and QOL remain unclear. This study aims to comprehensively assess the impact of ACSM-based exercise adherence on lung function and patient-reported QOL in adults with asthma.

**Methods:**

A systematic search of Cochrane, Web of Science, Embase, and PubMed was conducted to review a meta-analysis on exercise regimens with tailored prescriptions for symptomatic bronchial asthma patients. Eligible randomized controlled trials comparing exercise interventions to non-intervention were selected and analyzed using SMD and 95% CI. Study quality was assessed using the revised Cochrane Risk of Bias tool, while Egger’s regression and Begg’s test evaluated publication bias. Studies were classified based on adherence to ACSM guidelines, and subgroup analyses employed a random-effects model where appropriate to enhance result reliability and interpretability.

**Results:**

A total of 18 studies were included, with 9 classified as high adherence to ACSM guidelines and 9 as low/uncertain adherence. For FVC values were 0.72 (95% CI: 0.02, 1.42) and 0.64 (95% CI: 0.18, 1.11), respectively. The FEV1/FVC ratio was 0.19 (95% CI: −0.30, 0.69) versus 0.16 (95% CI: −0.95, 1.28). QOL scores demonstrated the most pronounced difference, with SMD at 0.85 (95% CI: 0.39, 1.32) for high adherence and 0.07 (95% CI: −0.22, 0.37) for low/uncertain adherence.

**Conclusion:**

This meta-analysis revealed that exercise interventions with high adherence to ACSM guidelines led to greater changes in QOL scores among asthma patients. While the high-adherence group outperformed the low/uncertain-adherence group in FEV_1_ and FVC, subgroup analysis failed to establish a significant difference. The modest impact on FEV_1_/FVC was likely influenced by substantial heterogeneity, potentially introducing bias in effect size estimation. Furthermore, the limited number of RCTs and small sample sizes may have undermined statistical power and result reliability.

**Systematic Review Registration:**

identifier CRD42024553618.

## 1 Introduction

Asthma is a chronic inflammatory disease marked by airway hyperresponsiveness and reversible airflow limitation, characterized by symptoms such as coughing, wheezing, shortness of breath, chest tightness, and dyspnea triggered by physical exertion, allergen exposure, or other factors, thereby limiting daily physical activity (Haselkorn et al., 2010). Asthma patients may also experience reduced physical fitness, along with comorbidities like depression, anxiety, and impaired quality of life (QOL) (Clark and Cochrane, 1988; Adams et al., 2001; Jenkins et al., 2005), which may further decrease adherence to medication (Turner et al., 2011) or lead to poor clinical control (Strine et al., 2008). As one of the most widespread chronic pulmonary disorder worldwide, the incidence and mortality of asthma continue to rise annually, with about 300 million people currently diagnosed with bronchial asthma worldwide, and causing around 1,000 deaths per day (Reddel et al., 2024). Healthcare expenditures for asthma patients account for about 1%–2% of total healthcare costs in industrialized countries (Ehteshami-Afshar et al., 2016). In addition to traditional pharmacological interventions like inhaled corticosteroids (ICS), exercise is now recommended as an effective non pharmacological strategy for asthma management (Reddel et al., 2024). As a key non-pharmacological intervention, exercise offers significant potential in alleviating the economic burden of asthma management. Previous studies have demonstrated that regular physical activity modulates immune function by reducing pro-inflammatory cytokine levels, an effect largely attributed to skeletal muscle—the most actively engaged organ during movement. Functioning as an endocrine organ, skeletal muscle secretes over 300 myokines, which play a crucial role in immune regulation (Giudice and Taylor, 2017; Afzali et al., 2018). Beyond its immunomodulatory effects, exercise also enhances pulmonary function by improving respiratory muscle strength and endurance, particularly in the diaphragm and intercostal muscles. This adaptation reduces the work of breathing, thereby optimizing ventilatory efficiency. Additionally, exercise-induced deep breathing and increased tidal volume mitigate alveolar atelectasis, improving the ventilation-perfusion ratio and enhancing systemic oxygenation (Neumann et al., 2005). Furthermore, exercise attenuates airway hyperresponsiveness by decreasing vagal nerve activity, promoting airway relaxation, and reducing bronchoconstriction. For individuals with asthma, formulating a well-structured exercise regimen—meticulously optimized in terms of intensity, frequency, and modality—is essential to maximizing therapeutic efficacy while ensuring safety. The primary objectives of such an intervention include alleviating symptoms, minimizing functional impairment, fostering greater participation in physical and social activities, and holistically improving health-related quality of life within the broader spectrum of respiratory disorders. Additionally, exercise may reduce dependence on healthcare resources and pharmacological treatments, particularly for asthma-associated comorbidities, including obesity. Previous studies have consistently demonstrated that nonadherence to asthma medication remains a widespread concern across the globe (Asamoah-Boaheng et al., 2021). Notably, the prevalence of poor adherence among asthma patients has been reported to reach 82.6% in Kuwait (Albassam et al., 2020), 86.1% in Northwestern Ethiopia (Belachew et al., 2022), 86% in Bangladesh (Rafi et al., 2022), 60.3% in Tanzania (Shayo et al., 2022), and as high as 88% in Northern Ireland (Gamble et al., 2009). Given the generally low adherence to conventional asthma medications, integrating exercise as an adjunctive strategy further reinforces its role as a critical component of comprehensive asthma management.

Exercise-induced dyspnea or the anticipation of exercise-related breathlessness often serves as a barrier, inhibiting asthma patients from engaging in physical activity, often resulting in poorer physical condition compared to their peers without asthma (Disabella and Sherman, 1998). Furthermore, physical inactivity detrimentally affects the social and emotional wellbeing of asthma patients, resulting in a diminished health-related quality of life (HRQOL). While exercise-induced bronchoconstriction can cause exercise intolerance in some asthma patients, the latest Global Initiative for Asthma (GINA) guidelines recognize exercise as an effective non-pharmacological strategy for enhancing asthma control (Reddel et al., 2024). A meta-analysis by Carson et al. concluded that patients with asthma show a high level of tolerance to exercise training (Carson et al., 2013). The British Thoracic Society guidelines on the physiotherapy supervision of adult asthma recommend that asthma patients engage in appropriate physical exercise to improve physical fitness, cardiopulmonary endurance, and HRQOL (Bott et al., 2009). These recommendations align with the GINA guidelines. Harika et al. (2014) and Ai Thi Hoang, (2015) demonstrated that yoga exercises significantly improve respiratory function in asthma patients, while Turner et al., (2011) found that regular physical exercise improves clinical symptoms and QOL scores in patients with fixed airway obstruction. However, no existing literature has comprehensively elucidated the optimal exercise frequency, intensity, and modality that yield the greatest improvements in pulmonary function and QOL scores in patients with asthma. The specific effects of different exercise protocols on pulmonary function parameters and QOL scores in this population remain inconclusive, necessitating further rigorous investigation.

The American College of Sports Medicine (ACSM) has established exercise prescription guidelines for generally healthy adults, encompassing comprehensive recommendations on the appropriate dosages of aerobic, resistance, and flexibility exercises tailored specifically for individuals with asthma (Garber et al., 2011). Nonetheless, it remains uncertain whether exercise interventions aligned with ACSM recommendations exert a more pronounced effect on pulmonary function parameters and QOL in asthma patients compared to those with low or uncertain adherence. The objective of this systematic review is to evaluate and compare the impact of exercise interventions with high adherence versus low or uncertain adherence to ACSM guidelines on pulmonary function and QOL outcomes in individuals with asthma.

## 2 Materials and methods

### 2.1 Search strategy

We conducted a search based on the PICOS principles in Cochrane, Web of Science, Embase, and PubMed databases from inception to 25 June 2024, focusing on study populations, interventions, and study methods. Search terms included: exercise, aerobic exercise, bronchial asthma, occupational asthma, and randomized controlled trials. The comprehensive search strategy is outlined in Supplementary Appendix SA1. Additionally, we performed manual backward citation searching through pertinent reviews and included studies. Using the “Related Articles” or “Similar Articles” functions in PubMed and Web of Science, a comprehensive search was conducted to identify relevant studies published before 25 June 2024. However, no articles meeting the predetermined criteria were found. The ACSM guidelines for exercise prescription, which specifically addressed asthma patients, were first published in 2011. Therefore, studies conducted prior to this year did not incorporate these specific exercise recommendations when designing their interventions. However, this does not compromise the validity of our literature search strategy or the reliability of the final results. The rationale lies in the fact that the 2011 edition of the ACSM’s Guidelines for Exercise Testing and Prescription (8th edition) was the first to explicitly outline exercise recommendations for asthma patients. Although subsequent editions, including those published in 2013 (9th edition), 2017 (10th edition), and 2021 (11th edition), introduced updates, the fundamental exercise recommendations for individuals with asthma have remained largely unchanged. Consequently, our search strategy did not exclude studies conducted prior to 2011. Although this study is based on the 2011 ACSM exercise guidelines, its primary objective is not to validate the efficacy of the guidelines themselves but rather to assess whether the included exercise interventions align with ACSM recommendations and to classify them according to adherence levels. To be precise, this study employs ACSM-recommended exercise protocols as the evaluation standard rather than using the year of guideline publication as a criterion for study selection. Thus, as long as the exercise interventions in a given study are consistent with or comparable to ACSM recommendations, studies conducted prior to the publication of the ACSM guidelines remain eligible for inclusion in our analysis. Therefore, the publication date of the ACSM guidelines does not restrict the time frame of our literature search.

### 2.2 Inclusion criteria for literature

We included studies that fulfilled the following criteria: (a) Published randomized controlled trials (RCTs); (b) Interventions encompassing any exercise regimen, such as resistance training, aerobic exercise, flexibility exercises, or similar modalities; (c) Participants diagnosed with bronchial asthma; (d) Reported outcomes related to pulmonary function parameters or QOL scores; (e) Control interventions consisting of either no treatment or any non-exercise-related intervention, thereby excluding studies that compared various exercise interventions.

We excluded studies based on the following criteria: (a) Reports, conference abstracts, and review articles; (b) Duplicate experimental data derived from multiple publications of the same study; (c) Studies in which participants received specific medications during the exercise intervention; (d) Research focused on aquatic exercise or studies that did not include a comparison between land-based exercise interventions and non-exercise control groups.

Two authors (JHL and JXZ) independently screened the titles and abstracts of the literature for eligibility. If either author determined that a study met the inclusion criteria, the full-text article was retrieved. Subsequently, both authors independently evaluated whether the full text met the study requirements. The inter-reviewer consistency in literature screening is evaluated using Cohen’s Kappa coefficient. A high Cohen’s Kappa value (>0.80) signifies strong concordance between reviewers, permitting the direct inclusion or exclusion of studies based on screening outcomes. Conversely, when the Cohen’s Kappa value is low (<0.60), the third author (JHW) is consulted to adjudicate discrepancies through discussion and render the final decision on study inclusion or exclusion, ensuring consensus. No restrictions were imposed based on participants’ age, gender, body mass index, publication date, or language. During the database search, we identified articles in French, German, and Portuguese. However, all of these studies were ultimately excluded as they did not meet the predetermined inclusion criteria.

### 2.3 Outcome measures

The primary outcome of this meta-analysis was the changes in pulmonary function, evaluated through changes in FEV_1_, FVC, and the FEV_1_/FVC ratio. These parameters were assessed using spirometry at baseline and follow-up.

Secondary outcomes included Asthma Quality of Life Questionnaire (AQLQ) and health-related quality of life (HRQoL), measured by the St. George’s Respiratory Questionnaire (SGRQ). Effect sizes were calculated using mean differences (MD) with 95% confidence intervals (95%CI) for continuous variables and risk ratios (RR) for categorical outcomes. However, all outcome measures in this study were continuous variables.

### 2.4 Data synthesis and analysis

Data extraction was performed independently by two authors (JHL and JXZ). The primary outcomes of the study were pulmonary function parameters and QOL scores. An Excel spreadsheet was pre-designed to capture relevant data attributes, including publication details (title, author names, year of publication), exercise characteristics (frequency, intensity, duration, repetitions, sets), methodological features (number of study groups, group design, intervention measures, sample size), risk assessment, and outcome data.

When extracting outcome data, if post-intervention results were not explicitly reported but were presented in graphical form, we employed Engauge Digitizer software to extract the specific data points. However, in the process of data extraction and result synthesis for the included studies, no digital visualization software was utilized for data processing. For studies with multiple follow-up assessments, only the immediate post-intervention data were extracted.

Upon completion of data extraction, we evaluated the exercise intervention dosages and adherence in the included studies according to the guidelines set forth by the American College of Sports Medicine (ACSM) for the development and maintenance of cardiopulmonary, muscular, skeletal, and neurological functions in patients with asthma (Garber et al., 2011). Two authors (JHL and BFW) independently evaluated the exercise interventions in each study according to ACSM guidelines, considering all aspects of exercise dosage, including frequency, intensity, and duration. They assigned scores based on predefined criteria to assess adherence to the recommended exercise dosage (Supplementary Table S1). The scoring range for each exercise parameter was from 0 to 2 points: a score of 2 indicated full adherence to the standard, 1 indicated uncertainty, and 0 indicated non-adherence. In cases of disagreement, the authors consulted with a third reviewer to reach a consensus. Utilizing this scoring system, we quantified the proportion of studies adhering to the ACSM-recommended exercise dosage. For each study, the actual score achieved was documented alongside the maximum attainable score to derive a compliance ratio. Based on the median values derived from previous literature, 70% was selected as the cutoff point to distinguish between high and low adherence levels (Cui et al., 2023; Cui et al., 2023; Wen et al., 2024). If this ratio was ≥70%, the study’s exercise intervention was categorized as exhibiting high adherence to the ACSM-prescribed exercise dosage (high ACSM adherence group). Conversely, if the ratio was <70%, the study was designated as having low or uncertain adherence to ACSM guidelines (low/uncertain ACSM adherence group).

### 2.5 Statistical analysis

We conducted a meta-analysis using STATA 16.0 to compare the outcomes of the included studies, which were categorized into two groups: the ACSM high-adherence group and the ACSM low/uncertain-adherence group. Additionally, to address the substantial heterogeneity, a subgroup analysis was conducted to assess whether variations in exercise modality—specifically aerobic versus resistance training—contributed to the observed discrepancies. Heterogeneity within each subgroup was assessed using the Higgins I^2^ statistic, which quantifies the proportion of total variation across studies attributable to heterogeneity rather than chance. Expressed as a percentage, a higher I^2^ value indicates greater inconsistency among study results and may necessitate further investigation into potential sources of heterogeneity. In accordance with the Cochrane Handbook guidelines (Deeks and Altman, 2011): heterogeneity was categorized as low (I^2^ ≤ 25%), moderate (25% < I^2^ ≤ 50%), substantial (50% < I^2^ ≤ 75%), or considerable (I^2^ > 75%). For heterogeneity assessment, a fixed-effects model was applied when I^2^ ≤ 50% to estimate the effect size, whereas a random-effects model was employed for I^2^ > 50%. Given the diversity in exercise protocols and intervention frequencies across the included studies, considerable clinical heterogeneity was evident. Therefore, a random-effects model was employed to ensure a more robust and generalized estimation of effect sizes. The effect size was quantified using the standardized mean difference (SMD) and its corresponding 95% confidence interval (95% CI), with the SMD classification adhering to Cohen’s effect size scale, where an SMD of 0.2 denotes a small effect, 0.5 reflects a moderate effect, 0.8 indicates a large effect, and values exceeding 1.0 signify a very large effect. A forest plot was employed to visually represent the central tendency and distribution of effect sizes across the included studies, thereby enhancing the interpretability of the results. All meta-analyses and forest plots were performed using Review Manager (RevMan) software, version 5.4. Confidence intervals were calculated using the default random-effects model (DerSimonian-Laird method with Wald-type confidence intervals), as implemented in RevMan 5.4. Statistical analysis was performed using the inverse variance weighting method to assign weights, ensuring that studies with higher precision contributed more substantially to the overall effect estimation. To evaluate publication bias, a funnel plot was constructed by plotting effect sizes against standard errors for each study. Asymmetry was formally assessed using Begg’s rank correlation test and Egger’s linear regression test, with a significance threshold of *p* < 0.05, indicating potential publication bias.

### 2.6 Quality assessment

The methodological quality of the included studies was evaluated by two pairs of authors (JHL and JXZ, JHW and BFW) using the quality assessment criteria endorsed by the Cochrane Collaboration (Higgins et al., 2011). All studies included in this review were randomized controlled trials. In accordance with the Cochrane Handbook, the Risk of Bias (ROB) tool is the recommended instrument for assessing the methodological rigor of randomized controlled trials (Sterne et al., 2019). This structured framework systematically evaluates bias across multiple outcomes in various types of randomized trials. Following Cochrane Handbook guidelines, reviewers categorize the risk of bias in each domain into three levels: “low risk,” “some concerns,” and “high risk,” ensuring a standardized and comprehensive assessment. If all domains are rated as “low risk”, the overall risk of bias is classified as “low”. If some domains are rated as “some concerns” and no domains are rated as “high risk”, the overall risk of bias is considered “some concerns”. If any domain is rated as “high risk” the overall risk of bias is classified as “high” (Cumpston et al., 2019). In addition to the assessment of risk of bias, we employed the GRADE (Grading of Recommendations Assessment, Development, and Evaluation) system to evaluate the certainty of the evidence in our meta-analysis. The GRADE framework considers factors such as study design, consistency of results, risk of bias, indirectness, and imprecision, providing a robust approach for assessing the overall confidence in the findings. This process was integral to ensuring the methodological rigor and transparency of our analysis.

The assessment criteria encompass random sequence generation, allocation concealment, blinding of participants and personnel, blinding of outcome assessment, incomplete outcome data, selective reporting, and other potential biases. To assess the inter-rater agreement between the two groups of reviewers regarding the risk of bias evaluation for the included studies, we employed Fleiss’s Kappa coefficient. When the Fleiss’s Kappa value was low (<0.6), indicating moderate or poor concordance, an additional independent reviewer (WZS) was introduced to adjudicate discrepancies and make the final determination on the risk of bias assessment.

### 2.7 Registration information

The systematic review and meta-analysis will be conducted and reported in compliance with the Preferred Reporting Items for Systematic Reviews and Meta-Analyses (PRISMA) guidelines (Page et al., 2021) and will be registered with PROSPERO (CRD42024553618).

## 3 Results

### 3.1 Literature selection

**FIGURE 1:**
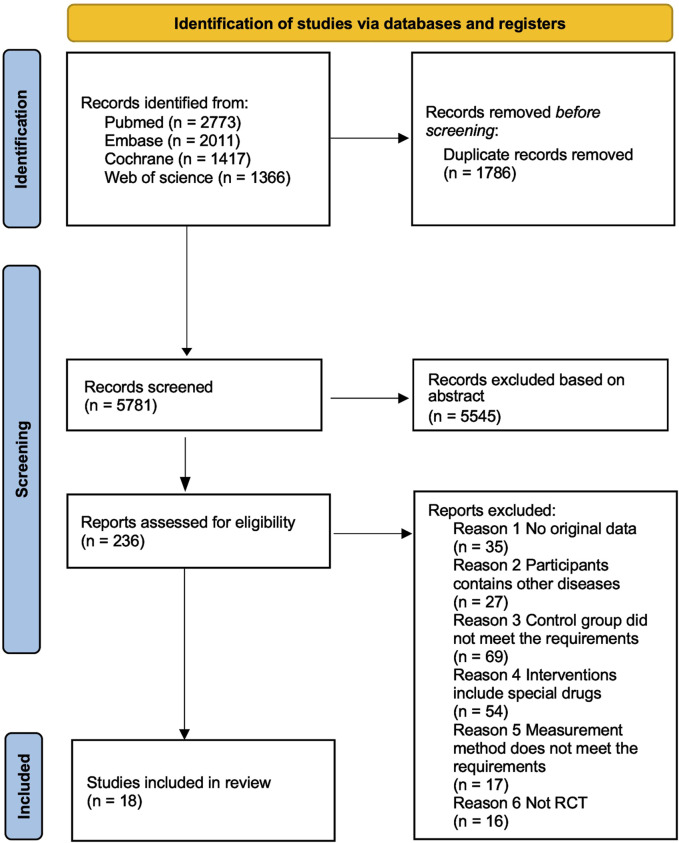
PRISMA study flow diagram.

A total of 7,567 articles were identified from 4 databases: PubMed (2,773), Embase (2,011), Web of Science (1,366), and Cochrane (1,417). After removing duplicates, 5,781 records were retained. Upon thorough review of titles and abstracts, 236 articles were considered as potential candidates. Finally, after comprehensive full-text review, 18 relevant articles were included (Farid et al., 2005; Mendes et al., 2010; Turner et al., 2010; Mendes et al., 2011; Scichilon et al., 2012; Scott et al., 2013; Arandelovic et al., 2015; Frana-Pinto et al., 2015; Meyer et al., 2015; Refaat and Gawish, 2015; Raghavendra et al., 2016; Toennesen et al., 2017; Coelho et al., 2018; Duruturk et al., 2018; Evaristo et al., 2020; Turan and Tan, 2020; Türk et al., 2020; Lage et al., 2021) (Figure1).

### 3.2 Article characteristics

The included studies consisted of 18 comparative trials, with a total of 876 participants, of whom 466 were assigned to the intervention group and 410 to the control group. In terms of gender distribution, the intervention group included 160 males and 306 females, while the control group comprised 128 males and 282 females. The participants’ ages ranged from 18 to 65 years. Geographically, six studies were conducted in Brazil, two in Turkey, two in Australia, and one each in Serbia, the Netherlands, Italy, Denmark, Egypt, India, Iran, and Germany. Participants were primarily recruited from hospital clinics, community settings, and through media advertisements (Supplementary Table S2).

The outcome measures from the included studies revealed that FEV1 was assessed in 14 studies, comprising 730 participants, with 385 in the intervention group and 345 in the control group; FVC was evaluated in 11 studies, involving 649 participants, with 340 in the intervention group and 309 in the control group; FEV1/FVC was measured in 6 studies, encompassing 381 participants, with 203 in the intervention group and 178 in the control group; and quality of life scores were reported in 12 studies, involving 538 participants, with 281 in the intervention group and 257 in the control group.

The intervention durations across the 18 studies varied from 6 weeks to 12 months, with exercise frequencies ranging from two to seven sessions per week. All studies incorporated either supervised or home-based exercise interventions. Among the 18 studies, 14 investigated aerobic exercise dosages, 5 examined resistance exercise dosages, and 8 focused on flexibility exercise dosages, as per the ACSM recommendations (Supplementary Table S3).

### 3.3 Risk of bias

**FIGURE 2:**
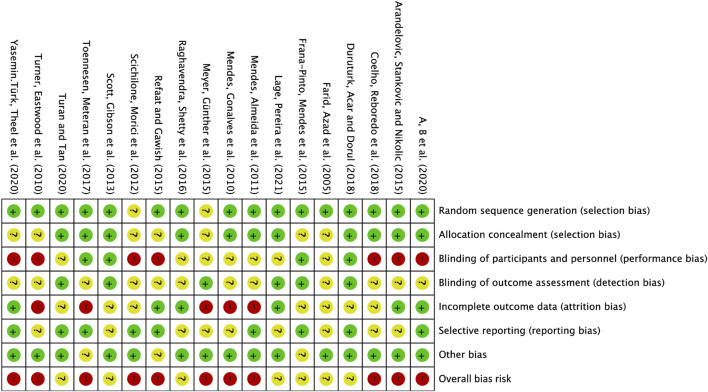
Risk of bias summary: The review author’s judgement of the risk of bias of each included study.

In the 18 studies included, 16 employed random sequence generation for group allocation, which is deemed to present a low risk of bias. Two studies did not explicitly indicate whether random allocation was used, and were therefore classified as having an uncertain risk of bias. Allocation concealment was assessed as low risk in 11 studies, while 7 studies did not provide details on the allocation method, resulting in an uncertain risk of bias. Given the inherent challenges of implementing double blinding in exercise interventions, 7 studies did not employ blinding for either researchers or participants, which is associated with a higher risk of bias. Another 7 studies did not clearly specify the use of blinding, thus categorizing them as having an uncertain risk of bias. Four studies explicitly reported blinding for both researchers and participants, considered to have a low risk of bias. Regarding outcome assessment blinding, 6 studies utilized random testing or blinded assessors, resulting in a low risk of bias, while 12 studies did not report the outcome assessment method, raising concerns and leading to an uncertain risk of bias. Six studies maintained a consistent number of participants from baseline to post-intervention, with complete outcome reporting, thus considered to have a low risk of bias. In 7 studies with incomplete outcome reporting, the post-intervention participant numbers were mostly consistent with baseline, which was classified as an uncertain risk of bias. Five studies exhibited a significant discrepancy in the number of participants pre- and post-intervention (≥10 participants), indicating a high risk of bias. Nine studies were rated low for selective reporting bias, whereas another 9 studies raised concerns due to the lack of pre-registered plans or insufficient explanations for participant withdrawals, leading to an uncertain risk of bias. Three studies presented an uncertain risk of other biases, mainly considering follow-up bias (Figure 2).

### 3.4 Adherence based on ACSM recommendations

Among the 18 studies, 9 demonstrated adherence to ACSM recommendations of ≥70%, while the remaining 9 had adherence levels below 70%. The primary factors contributing to low adherence included discrepancies between exercise intervention dosages and ACSM guidelines, as well as inadequate exercise prescription details for proper evaluation. When analysing adherence rates based on outcome measures: For studies assessing FEV1, 7 exhibited high adherence to ACSM recommendations, while 7 displayed low or uncertain adherence. For studies evaluating FVC, 5 showed high ACSM adherence, while 6 had low or uncertain adherence. Regarding FEV1/FVC as an outcome measure, 2 studies adhered to ACSM guidelines at a high level, while 4 had low or uncertain adherence. For studies reporting QOL scores, 7 studies adhered to ACSM recommendations with high fidelity, whereas 5 showed low or uncertain adherence.

### 3.5 Meta-analysis

#### 3.5.1 Pulmonary function test——FEV1

**FIGURE 3:**
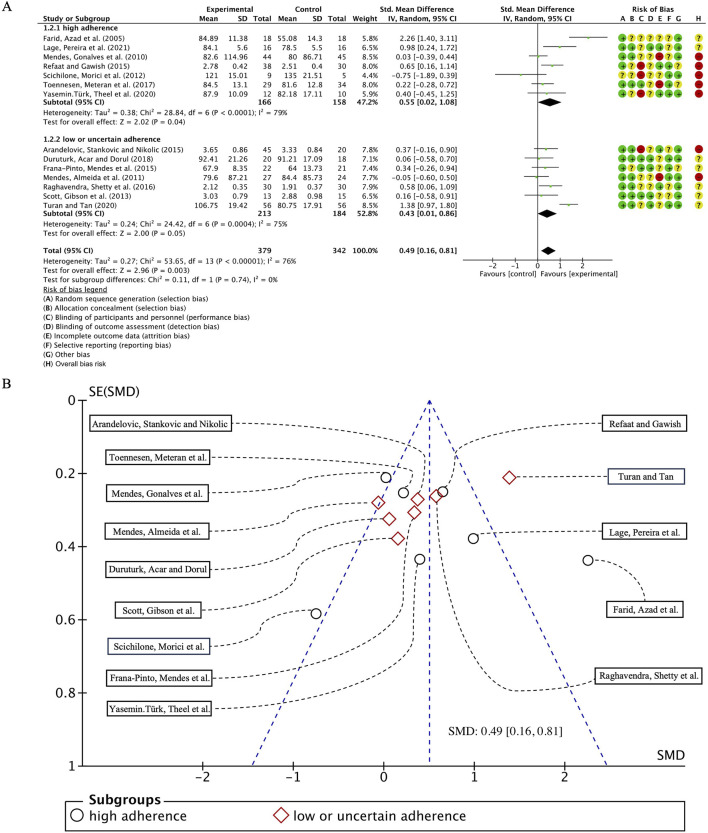
**(A)** Forest plot for meta-analyses of the effect of exercise on FEV1 in individuals of asthma. **(B)** Funnel plot containing FEV1 study.

In our analysis of 14 studies comprising 730 participants, with FEV1 as the primary outcome measure, we initially performed a heterogeneity assessment and observed that I^2^ exceeded 50% (I^2^ = 76%, *p* < 0.00001). Consequently, a random-effects model was employed for subsequent statistical analysis. The overall combined standardized mean difference (SMD) was 0.49 (95% CI: 0.16, 0.81), *p* = 0.003, indicating a significant beneficial effect of exercise interventions on FEV1 in individuals with bronchial asthma. In the subgroup analyses, studies were categorized according to adherence to ACSM recommendations. The pooled SMD for studies with high ACSM adherence was 0.55 (95% CI: 0.02, 1.08), *p* = 0.04, whereas for those with low or uncertain ACSM adherence, the combined SMD was 0.43 (95% CI: 0.01, 0.86), *p* = 0.05. However, no statistically significant difference was observed between the two subgroups, suggesting that adherence to ACSM guidelines does not confer a superior therapeutic advantage in improving FEV1 in patients with bronchial asthma (Figure 3A). Therefore, it can be concluded that exercise interventions with high adherence to ACSM guidelines do not yield superior therapeutic outcomes on FEV1 in bronchial asthma patients, compared to those with low or uncertain adherence.

Further analysis revealed that heterogeneity remained high within both subgroups, with I^2^ values of 79% and 75% for high and low/uncertain adherence groups, respectively. Assessment of publication bias using a funnel plot (Figure 3B) demonstrated near-symmetrical distribution, while statistical tests, including Begg’s test (*p* = 0.784) and Egger’s test (*p* = 0.814), confirmed the absence of significant publication bias.

**FIGURE 4:**
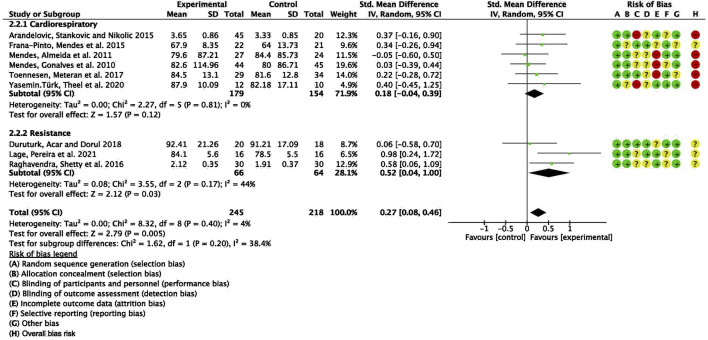
Forest plot for meta-analyses of the effect of exercise on FEV1 in individuals with asthma.

To explore potential sources of heterogeneity, an additional subgroup analysis was conducted based on exercise modality, comparing cardiorespiratory training with resistance training. The pooled SMD for studies with cardiorespiratory exercise was 0.18 (95% CI: −0.04, 0.39), *p* = 0.12, whereas for those with resistance exercise, the combined SMD was 0.52 (95% CI: 0.04, 1.00), *p* = 0.03. However, no statistically significant difference was detected between these subgroups (Figure 4).

#### 3.5.2 Pulmonary function test——FVC

**FIGURE 5:**
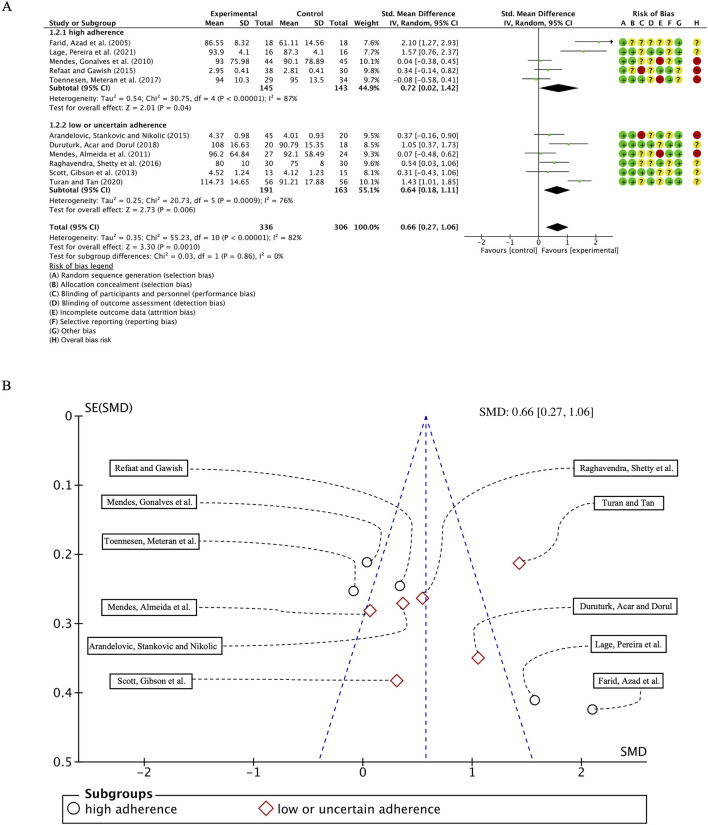
**(A)** Forest plot for meta-analyses of the effect of exercise on FEC in individuals with asthma. **(B)** Funnel plot containing FVC study.

In the 11 studies involving 649 participants with FVC as the outcome measure, we first conducted a heterogeneity test and found I^2^ to be greater than 50% (I^2^ = 82%, *p* < 0.00001); thus, a random-effects model was employed for statistical analysis. The results revealed an overall combined standardized mean difference (SMD) of 0.66 (95% CI: 0.27, 1.06), *p* = 0.0010, indicating a positive effect of exercise intervention on FVC in patients with bronchial asthma. Subgroup analyses based on adherence to ACSM recommendations showed that the combined SMD for the high ACSM adherence subgroup was 0.72 (95% CI: 0.02, 1.42), *p* = 0.04, while for the low or uncertain adherence subgroup, the combined SMD was 0.64 (95% CI: 0.18, 1.11), *p* = 0.006. Subgroup analysis revealed no statistically significant differences in FVC improvements between exercise interventions with high adherence to ACSM guidelines and those with low or uncertain adherence (Figure 5A). Consequently, it can be inferred that strict adherence to ACSM recommendations does not confer superior therapeutic benefits for FVC in patients with bronchial asthma.

Heterogeneity analysis within the subgroups indicated substantial variability among individual studies, with I^2^ values of 87% and 76% for the high and low/uncertain adherence groups, respectively. Funnel plot assessment (Figure 5B) demonstrated approximate symmetry, suggesting no notable publication bias. Furthermore, Begg’s test (*p* = 0.073) and Egger’s test (*p* = 0.249) corroborated this finding. However, the paucity of studies in the upper region of the funnel plot implies a potential lack of large-scale investigations addressing FVC outcomes.

Nonetheless, none of the 11 studies exclusively employed resistance training as an intervention. Consequently, a subgroup comparison between aerobic exercise and resistance training was not conducted to further explore potential sources of heterogeneity.

#### 3.5.3 Pulmonary function test–FEV1/FVC

**FIGURE 6:**
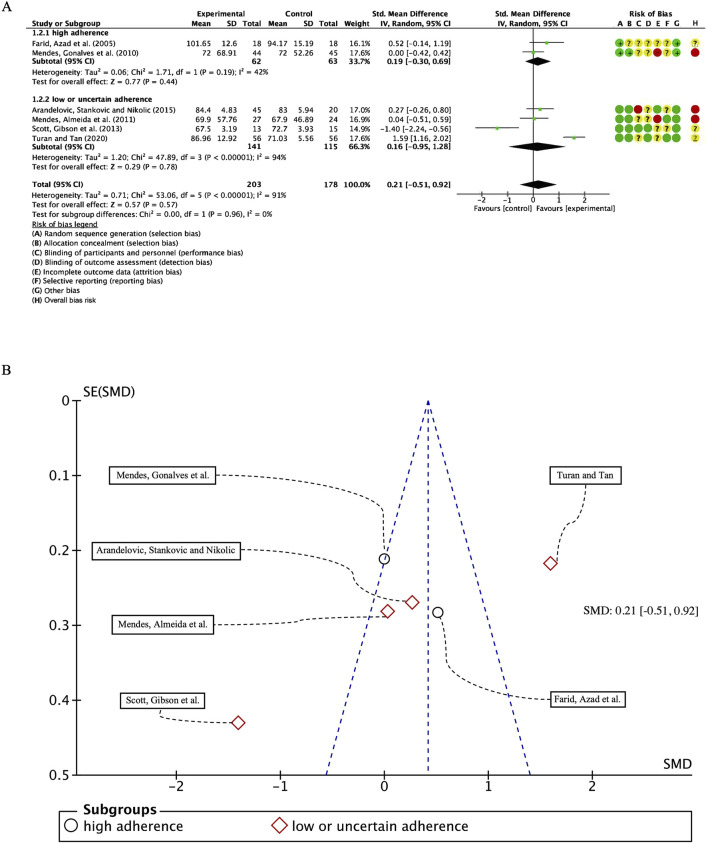
**(A)** Forest plot for meta-analyses of the effect of exercise on FEV1/FVC in individuals with asthma. **(B)** Funnel plot containing FEV1/FVC study.

In our analysis of six studies involving 381 participants with FEV1/FVC as the outcome measure, we first conducted a heterogeneity test, revealing I^2^ greater than 50% (I^2^ = 91%, *p* < 0.00001); consequently, we employed a random-effects model for statistical analysis. The overall combined standardized mean difference (SMD) was 0.21 (95% CI: −0.51, 0.92), *p* = 0.57, indicating no significant beneficial effect of exercise intervention on FEV1/FVC in patients with bronchial asthma. In the subgroup analyses, studies were categorized based on their adherence to ACSM guidelines. The combined SMD for the high ACSM adherence subgroup was 0.19 (95% CI: −0.30, 0.69), *p* = 0.44, while for the low or uncertain adherence subgroup, the combined SMD was 0.16 (95% CI: −0.95, 1.28), *p* = 0.78. Subgroup difference analysis revealed no significant distinctions between exercise interventions with high ACSM adherence and those with low or uncertain adherence (Figure 6A). Therefore, we conclude that exercise interventions with high ACSM adherence do not confer superior therapeutic effects on FEV1/FVC in patients with bronchial asthma relative to those with low or uncertain adherence.

In the subgroup with high ACSM adherence, the heterogeneity of individual studies for the outcome measure FEV1/FVC was 42%. In contrast, the subgroup with low or uncertain ACSM adherence exhibited a higher heterogeneity of 94%. The funnel plot (Figure 6B) demonstrated approximate symmetry, suggesting no substantial publication bias. Additionally, both the Begg’s test (*p* = 0.573) and the Egger’s test (*p* = 0.229) corroborated the absence of significant publication bias. Nevertheless the qualitative assessment of the funnel plots, the limited number of studies incorporated in each subgroup analysis, and the substantial interstudy heterogeneity appear to be incongruent with the reported findings. The field requires a substantial number of additional randomized controlled trials with adequately calibrated statistical power to rigorously confirm the nonexistence of publication bias within the existing body of scholarly literature.

**FIGURE 7:**
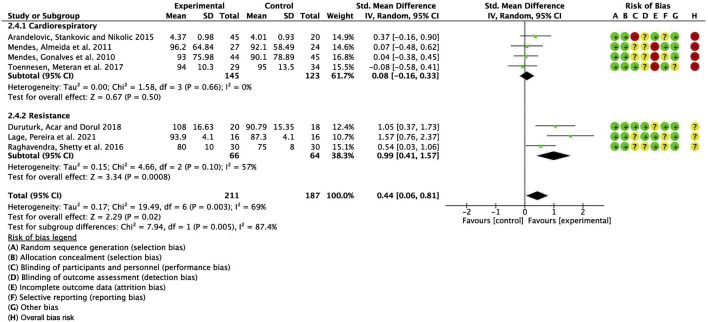
Forest plot for meta-analyses of the effect of exercise on FEV1/FVC in individuals with asthma.

To further investigate the potential sources of heterogeneity, we conducted an additional subgroup analysis, stratifying studies based on exercise modality (aerobic vs. resistance training) and comparing the differences between the two groups. The pooled SMD for studies with cardiorespiratory exercise was 0.08 (95% CI: −0.16, 0.33), *p* = 0.50, whereas for those with resistance exercise, the combined SMD was 0.99 (95% CI: 0.41, 1.57), *p* = 0.0008. The results revealed a statistically significant difference between these subgroups (Figure 7). Several factors may account for this observed discrepancy. First, categorizing studies according to exercise modality (aerobic vs. resistance training) may have introduced greater within-group heterogeneity, thereby amplifying the between-group differences compared to subgrouping based on ACSM adherence (high vs. low/uncertain). Second, the change in grouping criteria may have increased the influence of confounding variables, such as variations in exercise intensity and frequency, potentially exaggerating the observed between-group differences. Therefore, although the intergroup difference reached statistical significance, further research is warranted to ascertain its clinical relevance and to control for potential confounders, thereby enhancing the robustness of the findings.

#### 3.5.4 Quality of life score

**FIGURE 8:**
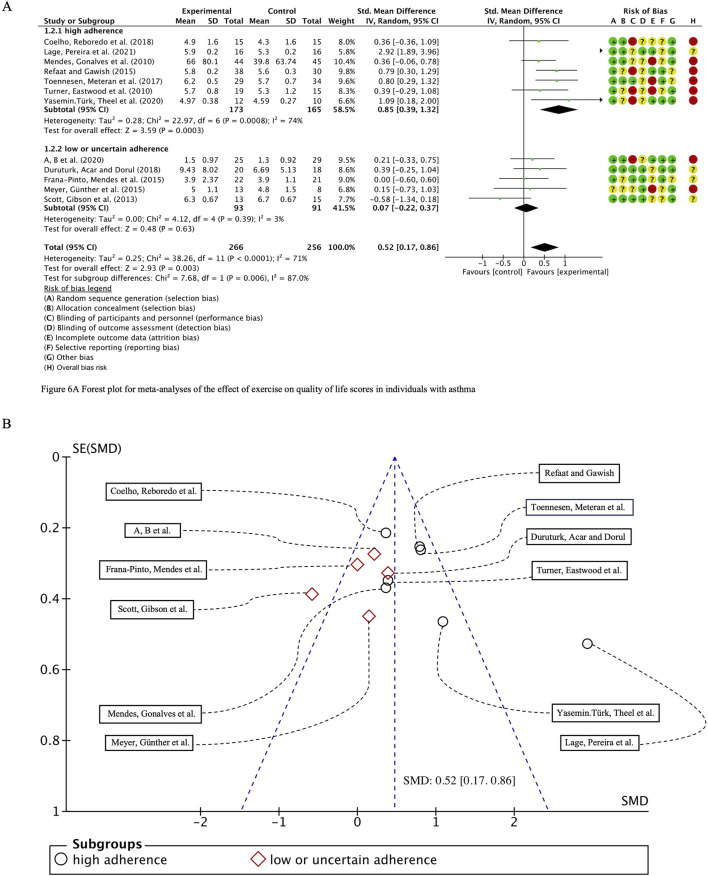
**(A)** Forest plot for meta-analyses of the effect of exercise on quality of life scores in individuals with asthma. **(B)** Funnel plot containing QOL study.

We systematically compiled key assessment metrics for evaluating the quality of life in asthma patients, including the QOL, HRQOL, SGRQ and AQLQ scores, and performed a comprehensive analysis encompassing 538 participants across 12 studies. Initial heterogeneity testing revealed substantial variability (I^2^ = 71%, *p* < 0.0001), prompting the use of a random-effects model for statistical analysis. The overall combined standardized mean difference (SMD) was 0.52 (95% CI: 0.17, 0.86), *p* = 0.003, indicating a positive effect of exercise interventions on the quality of life in individuals with bronchial asthma. Subgroup analyses stratified by ACSM adherence demonstrated that the pooled SMD for the high adherence subgroup was 0.85 (95% CI: 0.39, 1.32), *p* = 0.0003, whereas the pooled SMD for the low or uncertain adherence subgroup was 0.07 (95% CI: −0.22, 0.37), *p* = 0.63. However, subgroup difference analysis revealed no statistically significant discrepancy between high and low/uncertain adherence exercise interventions (Figure 8A). Therefore, it can be inferred that adherence to high ACSM standards does not confer superior benefits in improving the quality of life in asthma patients compared to interventions with low or uncertain adherence.

In the subgroup characterized by high ACSM adherence, the heterogeneity for the QOL score outcome measure was 74%, whereas in the subgroup with low or uncertain adherence, it was notably lower at 3%. Funnel plot analysis (Figure 8B) exhibited near symmetry, indicating no significant evidence of publication bias. This finding was further substantiated by the results of both the Begg test (*p* = 0.681) and the Egger test (*p* = 0.404), which confirmed the absence of substantial publication bias.

**FIGURE 9:**
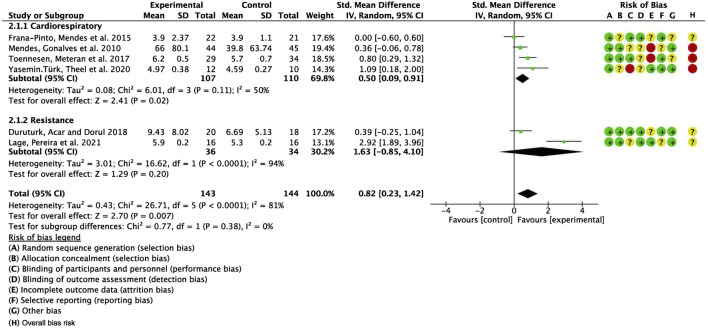
Forest plot for meta-analyses of the effect of exercise on QOL in individuals with asthma.

To further investigate potential origins of heterogeneity, a supplementary subgroup analysis was conducted based on exercise type, specifically evaluating aerobic training in comparison to resistance exercise. The pooled SMD for studies with cardiorespiratory exercise was 0.50 (95% CI: 0.09, 0.91), *p* = 0.02, whereas for those with resistance exercise, the combined SMD was 1.63 (95% CI: −0.85, 4.10), *p* = 0.20. Nevertheless, no significant statistical differences were identified between these categories (Figure 9).

#### 3.5.5 Certainty of evidence

The certainty of evidence was evaluated using the GRADE framework (Supplementary Table S5). The overall certainty was rated as high for FEV_1_ and QOL, indicating robust and reliable evidence supporting the beneficial effects of exercise interventions in these outcomes. In contrast, the certainty for FVC and FEV_1_/FVC was downgraded to moderate, primarily due to the presence of substantial heterogeneity and potential risk of bias in certain included studies. These factors introduce some degree of uncertainty, necessitating cautious interpretation of the findings. Further well-designed, high-quality randomized controlled trials (RCTs) are warranted to strengthen the evidence base and enhance the reliability of these conclusions.

## 4 Discussion

This article provides a comprehensive analysis of various exercise modes, intensity levels, durations, and other indicators used in previous studies. It explores the impact of exercise dosage grouped by ACSM adherence on pulmonary function indicators and quality of life scores in patients with bronchial asthma. Our study found that exercise intervention can improve FEV1 (SMD = 0.49; 95% CI: 0.16, 0.81), FVC (SMD = 0.66; 95% CI: 0.27, 1.06), and quality of life scores (SMD = 0.52; 95% CI: 0.17, 0.86) in patients with bronchial asthma. These results are consistent with previous research findings, indicating that exercise represents a potent non-pharmacological therapeutic approach for individuals with bronchial asthma (Dogra et al., 2011, Carson et al., 2013; Freitas et al., 2013; Frana-Pinto et al., 2015; Reddel et al., 2024). However, for FEV1/FVC (SMD = 0.21; 95% CI: −0.51, 0.92), the effect of exercise intervention is not significant. Subgroup analysis results show that, compared to the exercise intervention group with low or uncertain ACSM adherence, the exercise intervention with high ACSM adherence has better improvement effects on QOL scores (SMD 0.85 vs. 0.07) in patients with bronchial asthma. Nevertheless, the impact on FEV1 (SMD 0.55 vs. 0.43), FVC (SMD 0.72 vs. 0.64), and FEV1/FVC (SMD 0.19 vs. 0.16) remains negligible.

Through literature review, we found no other similar systematic reviews using ACSM adherence as a criterion to determine the impact of exercise dosage on patients with bronchial asthma. A substantial body of literature has confirmed the safety of exercise interventions in adult asthma patients. It also emphasizes the important role of sports as a non-pharmacological treatment method in complementing existing asthma management methods (Cordova-Rivera et al., 2018). Particularly for patients with moderate and severe asthma, sports are an important adjunct to drug treatment (Chandratilleke et al., 2005; Freitas et al., 2013; Reddel et al., 2024). It can not only improve health related quality of life (Dogra et al., 2011; Turner et al., 2011; Frana-Pinto et al., 2015), aerobic capacity (Cordova-Rivera et al., 2018), and clinical control (Dogra et al., 2011; Frana-Pinto et al., 2015), but also significantly alleviate anxiety and depression symptoms in asthma patients and reduce corticosteroid usage (Fanelli et al., 2007), alleviate psychosocial stress (Mendes et al., 2010), and mitigate dyspnea and alleviate asthma-related symptoms (Chandratilleke et al., 2005; Heikkinen et al., 2012; Carson et al., 2013; Mancuso et al., 2013).

When asthma patients engage in physical activities, the combination of exercise and respiratory training generates varying intra-abdominal pressures. Through such training, the strength and endurance of the diaphragm can be enhanced, thereby improving its function and, consequently, optimizing pulmonary function. Through diaphragmatic breathing, abnormal breathing patterns can be improved (Feng et al., 2020), increasing tidal volume, enhancing alveolar ventilation, reducing the work of breathing, and alleviating symptoms of dyspnea (Gosselink, 2003). Studies have shown that qigong exercises can enhance the immune system of the body and increase the CD4^+^ indicators in peripheral blood, thereby increasing FEV1, reducing airway inflammation, and improving the symptoms of asthma patients (Aijun et al., 2010). Yoga practice in asthma patients has been shown to increase tidal volume and forced vital capacity, reduce respiratory rate, and enhance pulmonary function (Makwana et al., 1988). Furthermore, yoga can improve the coordination of muscles, joints, and the entire musculoskeletal system, thereby strengthening the back, chest, and abdominal muscles, increasing breath depth, and enhancing pulmonary function (Vedanthan, 2003). Through the practice of controlled breathing techniques during yoga, respiratory endurance can be enhanced, resulting in increased oxygen uptake, improved pulmonary capacity, and a subsequent boost in overall vitality and stamina (Chanavirut et al., 2014).

Quality of life scores encompass the overall effects of disease and therapeutic interventions on patients’ physical functioning, psychological wellbeing, and social adaptation. We propose that the improvement in quality of life for asthma patients through traditional mind-body practices can be attributed to several factors: (a) The primary pathophysiological alteration in asthma is reversible obstructive ventilation dysfunction. The symptomatology during asthma exacerbations and the compromised pulmonary function in individuals with chronic asthma are significant determinants of quality of life. Through the practice of traditional mind-body exercises and controlled breathing techniques, patients enhance thoracic and pulmonary activity, expand chest volume, facilitate alveolar expansion, reduce respiratory muscle fatigue, and improve lung function, thus alleviating dyspnea and enhancing overall quality of life (Kupczyk and kuna, 2017; Hea Yon et al., 2020). (b) Long-term engagement in traditional mind-body exercises can substantially alleviate emotional states such as tension, anxiety, anger, hostility, and fatigue in patients (Yoshihara et al., 2011). (c) Simultaneously, many movements in traditional mind-body exercises necessitate collaborative participation, fostering interpersonal interactions. This serves to mitigate social isolation, alleviate feelings of emptiness and loneliness, regulate negative emotions, and facilitate the integration of patients into social networks.

A central objective of this study is to examine adherence to the ACSM guidelines. The exercise interventions recommended by the ACSM encompass aerobic exercise, resistance training, and flexibility exercises, with each modality specifying the requisite dosage. However, in randomized controlled trials involving patients with respiratory conditions, descriptions of exercise dosages are frequently incomplete or limited to specific intervention types. For example, eleven studies reported only the dosages for aerobic exercises, four for resistance training, and just five provided dosages in accordance with the full spectrum of ACSM-recommended categories. Additionally, some studies either failed to report or inadequately detailed the dosages of exercise interventions. This suggests that certain exercise interventions, despite being closely aligned with ACSM guidelines, may be misclassified as exhibiting low compliance or uncertain adherence. Similar to pharmacological treatments, a precise exercise prescription is essential for defining an appropriate range of dosages. While individualized treatment plans are necessary, adjustments should be made within the parameters of established exercise prescriptions.

This study has several limitations that may introduce potential biases in the results. Nine interventions, which align closely with ACSM guidelines, encompass a diverse range of exercise modalities, including balance training, resistance training, flexibility exercises, aquatic activities, and mind-body exercises. This diversity could introduce substantial heterogeneity across the studies. Furthermore, the interventions vary in terms of frequency, intensity, duration, and other parameters, which complicates direct comparisons and the establishment of universal standards for optimal exercise interventions. Previous meta-analyses and randomized controlled trials have often lacked comparative studies on exercise intensity and frequency, making it difficult to define and design the most effective exercise programs for patients with bronchial asthma. Additionally, all studies are susceptible to potential biases, with any unclear or high-risk factors potentially influencing the final estimates of intervention effects. The overall bias in this review is most likely associated with the blinding of both interventionists and participants, followed by the blinding of outcome assessment and reporting. Finally, despite efforts to reduce errors by extracting data from figures and tables, some inaccuracies remain unavoidable (Supplementary Table S4).

This study strictly included randomized controlled trials (RCTs) with consistent exercise intervention protocols, ensuring high homogeneity in both intervention measures and outcome indicators to facilitate cross-study horizontal comparisons. While this methodological approach enhances the comparability of results and mitigates the impact of methodological heterogeneity on research conclusions, several limitations persist, necessitating further refinement in future studies.

Firstly, this study did not incorporate direct within-study comparisons of distinct exercise modalities, thereby impeding a comprehensive evaluation of their relative efficacy in enhancing pulmonary function and improving quality of life scores. Specifically, the comparative advantages of aerobic exercise (e.g., endurance training) and respiratory muscle rehabilitation (e.g., inspiratory resistance training) over resistance training remain insufficiently elucidated due to the absence of systematic comparative analyses. Furthermore, this study did not explore exercise modalities that induce sustained alterations in respiratory dynamics, such as high-intensity interval training (HIIT) or breathing control exercises, whose potential superiority in optimizing pulmonary function parameters and augmenting quality-of-life scores remains uncertain. To address these gaps, future research should emphasize within-study comparative analyses of various exercise interventions, conducting direct assessments of the therapeutic efficacy of aerobic training, respiratory muscle rehabilitation, and resistance training to ascertain the most effective exercise intervention strategy for patients with pulmonary conditions.

Secondly, the physiological mechanisms mediating the beneficial effects of exercise interventions on pulmonary function in asthma patients remain insufficiently elucidated. This study primarily relied on conventional pulmonary function metrics and QOL scores as outcome measures, without probing into the underlying mechanistic pathways. For instance, whether exercise interventions mitigate airway hyperresponsiveness, enhance alveolar ventilation-perfusion efficiency, fortify respiratory musculature, or modulate immune-inflammatory cascades remains devoid of direct empirical substantiation. Future investigations should integrate comprehensive biomarker profiling (e.g., fractional exhaled nitric oxide [FeNO], sputum eosinophil counts, and peripheral blood cytokine signatures) alongside advanced respiratory mechanics assessments (e.g., lung compliance, airway resistance, and work of breathing) to construct a holistic framework delineating the physiological underpinnings of exercise-induced pulmonary benefits.

Finally, the exercise protocols employed in this study were relatively standardized, lacking stratified analyses based on disease progression, severity, and individual exercise tolerance, which may have contributed to the observed heterogeneity. Asthma patients exhibit substantial interindividual variability in terms of age, baseline physical fitness, pulmonary function status, and exercise capacity, rendering a uniform exercise intervention approach suboptimal. Therefore, future research should emphasize precision-tailored exercise prescriptions, integrating key patient-specific factors, such as age, disease phenotype, and exercise tolerance, while leveraging wearable technology for real-time physiological monitoring to optimize individualized intervention strategies. Furthermore, large-scale, high-quality RCTs are warranted to refine personalized exercise regimens, such as implementing high-intensity training paradigms for patients with mild-to-moderate asthma, while prescribing low-intensity, progressive exercise protocols for those with severe disease, thereby enhancing the clinical applicability and long-term adherence to exercise-based interventions.

## 5 Conclusion

This review reinforces the premise that exercise serves as an effective strategy for enhancing pulmonary function parameters and fundamental quality of life in individuals with bronchial asthma, with our findings providing further empirical support. In evaluating the optimal exercise dosage for this population, we identified that interventions adhering closely to ACSM guidelines yielded more pronounced improvements in QOL scores compared to those with low or uncertain adherence. However, subgroup analyses revealed no statistically significant differences in FEV1, FVC, or the FEV1/FVC, which may be attributed to the limited number of included studies, considerable heterogeneity within subgroups, the influence of multiple confounding variables, and an inherently high risk of bias. Moreover, the absence of detailed exercise intervention protocols in certain studies underscores the need for further validation in future research.

## Data Availability

The original contributions presented in the study are included in the article/Supplementary Material, further inquiries can be directed to the corresponding author.
