# Supplementary material for: The effects of exercise based on adherence to ACSM recommendations on pulmonary function and quality of life in adults with asthma: a systematic review and meta-analysis
Source: Front Physiol. 2025 May 15;16:1548382. doi: 10.3389/fphys.2025.1548382 (PMC12119264; doi:10.3389/fphys.2025.1548382)
Supplement: Supplementary file 1 [file DataSheet4.docx]

|  | Web of Science |
| --- | --- |
| #1 | ((((((((((((((((((((TS=(Asthma)) OR TS=(Occupational Asthma)) OR TS=(Exercise-Induced Cough-Variant Asthma)) OR TS=(asthma)) OR TS=(Asthma, Bronchial )) OR TS=(Bronchial Asthma)) OR TS=(asthma, Occupational)) OR TS=(Occupational Asthma)) OR TS=(Occupational asthma)) OR TS=(Asthma, Exercise Induced)) OR TS=(Exercise-Induced asthma)) OR TS=(Exercise-Induced Asthma)) OR TS=(Exercise Induced Asthma)) OR TS=(Bronchospasm, Exercise-Induced)) OR TS=(Bronchospasm, Exercise Induced)) OR TS=(Exercise-Induced Bronchospasms)) OR TS=(Exercise-Induced Bronchospasm)) OR TS=(Exercise Induced Bronchospasm)) OR TS=(Asthma, Cough-Variant)) OR TS=(Cough Variant Asthma)) OR TS=(Cough-Variant asthma) and Preprint Citation Index (Exclude – Database)  438923 |
| #2 | ((((((((((((((((((((((((((((((((TS=(Exercise)) OR TS=(Circuit-Based Exercise)) OR TS=(Exercises)) OR TS=(Exercise, Physical)) OR TS=(Exercises, Physical)) OR TS=(Physical Exercise)) OR TS=(Physical Exercises)) OR TS=(Physical Activity)) OR TS=(Activities, Physical)) OR TS=(Activity, Physical)) OR TS=(Physical Activities)) OR TS=(Exercise, Aerobic)) OR TS=(Aerobic Exercise)) OR TS=(Aerobic Exercises)) OR TS=(Exercises, Aerobic)) OR TS=(Exercise, Isometric)) OR TS=(Exercises, Isometric)) OR TS=(Isometric Exercises)) OR TS=(Isometric Exercise)) OR TS=(Acute Exercise)) OR TS=(Acute Exercises)) OR TS=(Exercise, Acute)) OR TS=(Exercises, Acute)) OR TS=(Exercise Training)) OR TS=(Exercise Trainings)) OR TS=(Training, Exercise)) OR TS=(Trainings, Exercise)) OR TS=(Circuit Based Exercise)) OR TS=(Circuit-Based Exercises)) OR TS=(Exercise, Circuit-Based)) OR TS=(Exercises, Circuit-Based)) OR TS=(Circuit Training)) OR TS=(Training, Circuit) and Preprint Citation Index (Exclude – Database)  2362750 |
| #3 | #1 AND #2  1366 |
