# Supplementary material for: The effects of exercise based on adherence to ACSM recommendations on pulmonary function and quality of life in adults with asthma: a systematic review and meta-analysis
Source: Front Physiol. 2025 May 15;16:1548382. doi: 10.3389/fphys.2025.1548382 (PMC12119264; doi:10.3389/fphys.2025.1548382)
Supplement: Supplementary file 2 [file Table1.docx]

Table 1 ACSM exercise recommendations for asthma patients

| Exercise dose | Cardiorespiratory exercise | Resistance exercise | Flexibility exercise |
| --- | --- | --- | --- |
| Frequency | 3 days per week | 2–3 days per week | More effective on ≥ 5 days per week, daily |
| Intensity/workload | 40–60% VO^2^R or HRR; RPE of 12–13 on a 6–20 scale | Start with 40–50% 1RM, more capable with 60–70% 1RM | Full range of flexion, extension and rotation |
| Duration | Continuous or cumulative 30 min | ≥ 1 group, 8–12 repetitions; adult Asthma's patients started with 10–15 repetitions | Keep static pulling for 10–30s; repeat 2–4 times |

HRR heart rate reserve, VO^2^R oxygen uptake reserve, RPE rating of perceived exertion, 1RM one repetition maximum
