# Supplementary material for: The effects of exercise based on adherence to ACSM recommendations on pulmonary function and quality of life in adults with asthma: a systematic review and meta-analysis
Source: Front Physiol. 2025 May 15;16:1548382. doi: 10.3389/fphys.2025.1548382 (PMC12119264; doi:10.3389/fphys.2025.1548382)
Supplement: Supplementary file 3 [file DataSheet3.docx]

|  | Embase |
| --- | --- |
| #1 | 'asthma'/exp OR asthma OR ('exercise induced' AND 'cough variant' AND asthma) OR asthmas OR (asthma, AND bronchial) OR (bronchial AND asthma) OR (asthmas, AND occupational) OR (occupational AND asthma) OR (occupational AND asthmas) OR (asthma, AND exercise AND induced) OR ('exercise induced' AND asthmas) OR ('exercise induced' AND asthma) OR (exercise AND induced AND asthma) OR (bronchospasm, AND 'exercise induced') OR (bronchospasm, AND exercise AND induced) OR ('exercise induced' AND bronchospasms) OR ('exercise induced' AND bronchospasm) OR (exercise AND induced AND bronchospasm) OR (asthma, AND 'cough variant') OR (cough AND variant AND asthma) OR ('cough variant' AND asthmas)  400212 |
| #2 | 'exercise'/exp OR exercise OR ('circuit based' AND exercise) OR exercises OR (exercise, AND physical) OR (exercises, AND physical) OR (physical AND exercise) OR (physical AND exercises) OR (physical AND activity) OR (activities, AND physical) OR (activity, AND physical) OR (physical AND activities) OR (exercise, AND aerobic) OR (aerobic AND exercise) OR (aerobic AND exercises) OR (exercises, AND aerobic) OR (exercise, AND isometric) OR (exercises, AND isometric) OR (isometric AND exercises) OR (isometric AND exercise) OR (acute AND exercise) OR (acute AND exercises) OR (exercise, AND acute) OR (exercises, AND acute) OR (exercise AND training) OR (exercise AND trainings) OR (training, AND exercise) OR (trainings, AND exercise) OR (circuit AND based AND exercise) OR ('circuit based' AND exercises) OR (exercise, AND 'circuit based') OR (exercises, AND 'circuit based') OR (circuit AND training) OR (training, AND circuit)  1220448 |
| #3 | #1 AND #2  2011 |
