# Supplementary material for: The effects of exercise based on adherence to ACSM recommendations on pulmonary function and quality of life in adults with asthma: a systematic review and meta-analysis
Source: Front Physiol. 2025 May 15;16:1548382. doi: 10.3389/fphys.2025.1548382 (PMC12119264; doi:10.3389/fphys.2025.1548382)
Supplement: Supplementary file 4 [file Table2.docx]

Table 2 Study characteristics

| Reference | Country | Initial sample size | | Gender Ratio | | | | Age(year) | | Outcome measure |
| --- | --- | --- | --- | --- | --- | --- | --- | --- | --- | --- |
|  |  | Ig | Cg | Ig(M) | Ig(F) | Cg(M) | Cg(F) | Ig | Cg |  |
| Turan and Tan (2020) | Turkey | 56 | 56 | 7 | 49 | 11 | 45 | 37.43 ± 9.58 | 40.28 ± 9.23 | FEV1, FVC, FEV1/FVC |
| Arandelovic, Stankovic and Nikolic (2015) | Serbia | 45 | 20 | 34 | 11 | 15 | 5 | 33.07 ± 9.81 | 33.55 ± 10.88 | FEV1, FVC, FEV1/FVC |
| Yasemin.Türk, Theel et al. (2020) | Netherlands | 14 | 10 | 4 | 10 | 1 | 9 | 41.57 ± 9.73 | 41.90 ± 8.58 | FEV1, QOL |
| Turner, Eastwood et al. (2010) | Australia | 19 | 15 | 8 | 11 | 7 | 8 | 65.38 ± 10.8 | 71.08 ± 9.7 | QOL |
| Lage, Pereira et al. (2021) | Brazil | 20 | 19 | 6 | 14 | 4 | 15 | 40.25 ± 13.4 | 42.26 ± 12.6 | FEV1, FVC, QOL |
| Scichilone, Morici et al. (2012) | Italy | 9 | 5 | 3 | 6 | 1 | 4 | 24 ± 2.1 | 26 ± 3.2 | FEV1 |
| Toennesen, Meteran et al. (2017) | Denmark | 29 | 34 | 16 | 13 | 8 | 26 | 39.4 ± 12.5 | 38.2 ± 12.7 | FEV1, FVC, QOL |
| Coelho, Reboredo et al. (2018) | Brazil | 20 | 17 | 2 | 18 | 3 | 14 | 45 ± 19 | 47 ± 14 | QOL |
| A, B et al. (2020) | Brazil | 29 | 25 | 7 | 22 | 8 | 17 | 49.8 ± 9.7 | 50.6 ± 9.2 | QOL |
| Mendes, Gonalves et al. (2010) | Brazil | 44 | 45 | 5 | 39 | 10 | 35 | 39 ± 42.6 | 39.5 ± 39.1 | FEV1, FVC, FEV1/FVC, QOL |
| Refaat and Gawish (2015) | Egypt | 38 | 30 | 17 | 21 | 14 | 16 | 35.8 ± 1.7 | 38 ± 5.3 | FEV1, FVC, QOL |
| Mendes, Almeida et al. (2011) | Brazil | 27 | 24 | 3 | 24 | 6 | 18 | 37.9 ± 27.3 | 36 ± 30.2 | FEV1, FVC, FEV1/FVC, |
| Duruturk, Acar and Dorul (2018) | Turkey | 20 | 18 | 6 | 14 | 1 | 17 | 46.50 ± 13.38 | 42.72 ± 18.85 | FEV1, FVC, QOL |
| Raghavendra, Shetty et al. (2016) | India | 30 | 30 | 18 | 12 | 17 | 13 | 31.80 ± 8.47 | 31.10 ± 8.11 | FEV1, FVC |
| Farid, Azad et al. (2005) | Iran | 18 | 18 | 8 | 10 | 8 | 10 | 27 | 29 | FEV1, FVC, FEV1/FVC |
| Scott, Gibson et al. (2013) | Australia | 13 | 15 | 6 | 7 | 7 | 8 | 33.9 ± 11.5 | 44.7 ± 14.7 | FEV1, FVC, FEV1/FVC, QOL |
| Frana-Pinto, Mendes et al. (2015) | Brazil | 22 | 21 | 5 | 17 | 4 | 17 | 40 ± 11 | 44 ± 9 | FEV1, QOL |
| Meyer, Günther et al. (2015) | Germany | 13 | 8 | 5 | 8 | 3 | 5 | 54 ± 11 | 59 ± 9 | QOL |

Numbers are mean (SD) unless otherwise stated. IG, Intervention group; CG, Control group; F:M, Female: Male
