# Supplementary material for: The effects of exercise based on adherence to ACSM recommendations on pulmonary function and quality of life in adults with asthma: a systematic review and meta-analysis
Source: Front Physiol. 2025 May 15;16:1548382. doi: 10.3389/fphys.2025.1548382 (PMC12119264; doi:10.3389/fphys.2025.1548382)
Supplement: Supplementary file 5 [file Table5.docx]

Table 5 The GRADE certainty of evidence

| Certainty assessment | | | | | | | | Number of participants | | Effect | | Certainty | Outcome measures |  |
| --- | --- | --- | --- | --- | --- | --- | --- | --- | --- | --- | --- | --- | --- | --- |
| Number of studies included | Research Type | | Risk of bias | Discrepancy | Indirectness | Accuracy | Other factors | interventions | placebo | Comparative (95% CI) | Absolute (95% CI) |  |  |  |
|  | |  | | | | | | | | | | | | |
| 14 | RCTs | | Non-serious | Non-serious | Non-serious | Non-serious | None | 379 | 342 | - | SMD: 0.49  Higher | ⨁⨁⨁⨁ High | FEV1 |  |
| 11 | RCTs | | Non-serious | Serious | Non-serious | Non-serious | None | 336 | 306 | - | SMD 0.66 Higher | ⨁⨁⨁◯ Moderate | FVC |  |
| 6 | RCTs | | Serious | Non-serious | Non-serious | Non-serious | None | 203 | 205 | - | SMD 0.21 Higher | ⨁⨁⨁◯ Moderate | FEV1/FVC |  |
| 12 | RCTs | | Non-serious | Non-serious | Non-serious | Non-serious | None | 266 | 256 | - | SMD 0.52 Higher | ⨁⨁⨁⨁ High | QOL |  |
