# Supplementary material for: The effects of exercise based on adherence to ACSM recommendations on pulmonary function and quality of life in adults with asthma: a systematic review and meta-analysis
Source: Front Physiol. 2025 May 15;16:1548382. doi: 10.3389/fphys.2025.1548382 (PMC12119264; doi:10.3389/fphys.2025.1548382)
Supplement: Supplementary file 6 [file Table3.docx]

Table 3 Characteristics of the study intervention

| Author, year | Interventions | Length of intervention | FEV1 | FVC | FEV1/FVC | Quality of life score |
| --- | --- | --- | --- | --- | --- | --- |
| Turan and Tan (2020) | Yoga | 6 weeks | Liter | Liter | ratio |  |
| Arandelovic, Stankovic and Nikolic (2015) | Practice swimming and treat with low dosage ICS and short-acting β2 agonists salbutamol | 6 months | Liter/second | Liter | ratio |  |
| Yasemin.Türk, Theel et al. (2020) | Pulmonary rehabilitation, including exercise training, nutritional intervention and psychological group sessions | 12 weeks | %pred |  |  | AQLQ |
| Turner, Eastwood et al. (2010) | Warm-up, 20-min walking, upper limb endurance training | 6 weeks |  |  |  | AQLQ |
| Lage, Pereira et al. (2021) | inspiratory muscle training | 8 weeks | Liter | Liter |  | AQLQ |
| Scichilone, Morici et al. (2012) | indoor rowing training | 10 weeks | %pred |  |  |  |
| Toennesen, Meteran et al. (2017) | high-intensity interval training | 8 weeks | %pred | %pred |  | AQLQ |
| Coelho, Reboredo et al. (2018) | moderate-intensity walk | 12 weeks |  |  |  | AQLQ |
| A, B et al. (2020) | indoor treadmill | 3 months |  |  |  | AQLQ |
| Mendes, Gonalves et al. (2010) | aerobic training program | 3 months | %pred | %pred | ratio | HRQOL |
| Refaat and Gawish (2015) | warm-up, stretching and exercise circuit | 6 weeks | %pred | %pred |  | AQLQ |
| Mendes, Almeida et al. (2011) | aerobic training | 3 months | %pred | %pred | ratio |  |
| Duruturk, Acar and Dorul (2018) | inspiratory muscle training | 6 weeks | Liter | Liter |  | SGRQ |
| Raghavendra, Shetty et al. (2016) | yoga(Kapalabhati) | NR | Liter | Liter |  |  |
| Farid, Azad et al. (2005) | aerobic exercise | 8 weeks | NR | NR | NR |  |
| Scott, Gibson et al. (2013) | aerobic activities and resistance training | 12 weeks | %pred | %pred | ratio | AQLQ |
| Frana-Pinto, Mendes et al. (2015) | aerobic training | 12 weeks | %pred |  |  | AQLQ |
| Meyer, Günther et al. (2015) | circuit training and stretch | 12 months |  |  |  | AQLQ |

NR, Not Reported. %pred, percentage of prediction value. SGRQ, St George’s Respiratory Questionnaire.
