# Supplementary material for: The effects of exercise based on adherence to ACSM recommendations on pulmonary function and quality of life in adults with asthma: a systematic review and meta-analysis
Source: Front Physiol. 2025 May 15;16:1548382. doi: 10.3389/fphys.2025.1548382 (PMC12119264; doi:10.3389/fphys.2025.1548382)
Supplement: Supplementary file 7 [file DataSheet2.docx]

|  | Pubmed |
| --- | --- |
| #1 | ((Asthma[MeSH Terms]) OR (Occupational Asthma[MeSH Terms])) OR (Exercise-Induced Cough-Variant Asthma[MeSH Terms]) Sort by: Most Recent 146009 |
| #2 | ((((((((((((((((((((Asthma) OR (Occupational Asthma)) OR (Exercise-Induced Cough-Variant Asthma)) OR (Asthmas)) OR (Asthma, Bronchial)) OR (Bronchial Asthma)) OR (Asthmas, Occupational)) OR (Occupational Asthma)) OR (Occupational Asthmas)) OR (Asthma, Exercise Induced)) OR (Exercise-Induced Asthmas)) OR (Exercise-Induced Asthma)) OR (Exercise Induced Asthma)) OR (Bronchospasm, Exercise-Induced)) OR (Bronchospasm, Exercise Induced)) OR (Exercise-Induced Bronchospasms)) OR (Exercise-Induced Bronchospasm)) OR (Exercise Induced Bronchospasm)) OR (Asthma, Cough-Variant)) OR (Cough Variant Asthma)) OR (Cough-Variant Asthmas) Sort by: Most Recent  225340 |
| #3 | Search: (#1) OR (#2) Sort by: Most Recent  225340 |
| #4 | (Exercise[MeSH Terms]) OR (Circuit-Based Exercise[MeSH Terms]) Sort by: Most Recent  258809 |
| #5 | ((((((((((((((((((((((((((((((((Exercise) OR (Circuit-Based Exercise)) OR (Exercises)) OR (Exercise, Physical)) OR (Exercises, Physical)) OR (Physical Exercise)) OR (Physical Exercises)) OR (Physical Activity)) OR (Activities, Physical)) OR (Activity, Physical)) OR (Physical Activities)) OR (Exercise, Aerobic)) OR (Aerobic Exercise)) OR (Aerobic Exercises)) OR (Exercises, Aerobic)) OR (Exercise, Isometric)) OR (Exercises, Isometric)) OR (Isometric Exercises)) OR (Isometric Exercise)) OR (Acute Exercise)) OR (Acute Exercises)) OR (Exercise, Acute)) OR (Exercises, Acute)) OR (Exercise Training)) OR (Exercise Trainings)) OR (Training, Exercise)) OR (Trainings, Exercise)) OR (Circuit Based Exercise)) OR (Circuit-Based Exercises)) OR (Exercise, Circuit-Based)) OR (Exercises, Circuit-Based)) OR (Circuit Training)) OR (Training, Circuit) Sort by: Most Recent  820991 |
| #6 | Search: (#4) OR (#5) Sort by: Most Recent  820991 |
| #7 | Search: (#3) AND (#6) Sort by: Most Recent  2773 |
