# Supplementary material for: The effects of exercise based on adherence to ACSM recommendations on pulmonary function and quality of life in adults with asthma: a systematic review and meta-analysis
Source: Front Physiol. 2025 May 15;16:1548382. doi: 10.3389/fphys.2025.1548382 (PMC12119264; doi:10.3389/fphys.2025.1548382)
Supplement: Supplementary file 8 [file Table4.docx]

Table 4 Exercise interventions evaluated according to the American College of Sports Medicine s (ACSM) recommendations

| Author, year | Cardiorespiratory exercise | | | | | | | | | Resistance exercise | | | | | | | | | | | | | Flexibility exercise | | | | | | | | | ACSM compliance | |
| --- | --- | --- | --- | --- | --- | --- | --- | --- | --- | --- | --- | --- | --- | --- | --- | --- | --- | --- | --- | --- | --- | --- | --- | --- | --- | --- | --- | --- | --- | --- | --- | --- | --- |
|  | Frequency  Days/week | | | Intensity/  workload | | | | Duration  (min) | | Frequency  Days/week | | | Intensity/  workload | | | Repetition  (min) | | | Set  (group) | | | | Frequency  Days/week | | Intensity/  workload | | | Duration  (min) | | | | Points/  Percent | |
| Turan and Tan (2020) |  | | |  | | | |  | |  | | |  | | |  | | |  | | | | 2 | ☹ | NR | | 😐 | 70 | | | ☺ | 3/6 | 50% |
| Arandelovic, Stankovic and Nikolic (2015) | 2 | | 😐 | NR | | 😐 | | 60 | ☺ |  | | |  | | |  | | |  | | | |  | |  | | |  | | | | 4/6 | 67% |
| Yasemin.Türk, Theel et al. (2020) | 3 | | ☺ | 90% VO2max | | ☺ | | 40-60 | ☺ |  | | |  | | |  | | |  | | | |  | |  | | |  | | | | 6/6 | 100% |
| Turner, Eastwood et al. (2010) | 3 | | ☺ | NR | | 😐 | | 30 | ☺ | 3 | ☺ | | Brog 6-20 RPE scale 12-14 | ☺ | | 45 | ☺ | | NR | | | 😐 | 3 | 😐 | Full-body stretches | | ☺ | 10-15 | | | ☺ | 17/20 | 85% |
| Lage, Pereira et al. (2021) |  | | |  | | | |  | | 5 | ☺ | | 50% of maximal inspiratory pressur | 😐 | | NR | 😐 | | 6 | | | ☺ |  | |  | | |  | | | | 6/8 | 75% |
| Scichilone, Morici et al. (2012) | 2-3 | ☺ | | NR | 😐 | | | 70 | ☺ |  | | |  | | |  | | |  | | | | 2-3 | 😐 | Stretch | | 😐 | 20 | | ☺ | | 9/12 | 75% |
| Toennesen, Meteran et al. (2017) | 3 | ☺ | | NR | 😐 | | | 20-30 | ☺ |  | | |  | | |  | | |  | | | |  | |  | | |  | | | | 5/6 | 83% |
| Coelho, Reboredo et al. (2018) | 5 | ☺ | | NR | 😐 | | | 30-90 | ☺ |  | | |  | | |  | | |  | | | | 5 | ☺ | Stretch | | ☺ | NR | 😐 | | | 10/12 | 83% |
| A, B et al. (2020) | 2 | 😐 | | NR | 😐 | | | 35 | ☺ |  | | |  | | |  | | |  | | | | 2 | ☹ | Stretch | | 😐 | 5 | 😐 | | | 6/12 | 50% |
| Mendes, Gonalves et al. (2010) | 2 | 😐 | | 60-70% VO2max | ☺ | | | 30 | ☺ |  | | |  | | |  | | |  | | | |  | |  | | |  | | | | 5/6 | 83% |
| Refaat and Gawish (2015) | 3 | ☺ | | 60-80%  MHR | ☺ | | | 20-30 | ☺ |  | | |  | | |  | | |  | | | | 3 | 😐 | Stretch | 😐 | | 10 | ☺ | | | 10/12 | 83% |
| Mendes, Almeida et al. (2011) | 2 | 😐 | | NR | 😐 | | | 30 | ☺ |  | | |  | | |  | | |  | | | |  | |  | | |  | | | | 4/6 | 67% |
| Duruturk, Acar and Dorul (2018) |  | | |  | | | |  | | 7 | | ☺ | 50% maximal inspiratory pressure | 😐 | | NR | | 😐 | 2 | | 😐 | |  | |  | | |  | | | | 5/8 | 63% |
| Raghavendra, Shetty et al. (2016) |  | | |  | | | |  | | NR | | 😐 | NR | 😐 | | 10 | | ☺ | NR | | 😐 | |  | |  | | |  | | | | 5/8 | 62.5% |
| Farid, Azad et al. (2005) | 3 | ☺ | | NR | | 😐 | | 20 | ☺ |  | | |  | | |  | | |  | | | | 3 | 😐 | Stretch | 😐 | | 15 | ☺ | | | 9/12 | 75% |
| Scott, Gibson et al. (2013) | 3 | ☺ | | NR | | 😐 | | NR | 😐 | 3 | | ☺ | NR | | 😐 | NR | | 😐 | NR | 😐 | | |  | |  | | |  | | | | 9/14 | 64% |
| Frana-Pinto, Mendes et al. (2015) | 2 | 😐 | | NR | | | 😐 | 35 | ☺ |  | | |  | | |  | | |  | | | |  | |  | | |  | | | | 4/6 | 67% |
| Meyer, Günther et al. (2015) | 1 | ☹ | | 60%  MHR | | | ☺ | 45 | ☺ |  | | |  | | |  | | |  | | | | 1 | ☹ | NR | 😐 | | 15 | ☺ | | | 7/12 | 58% |

ACSM, American College of Sports Medicine. NR, not reported. Happy/green face, fulfils recommendation (2 points), neutral/yellow face, uncertain fulfilment (1 point), unhappy/red face, does not fulfil recommendation (0 point).
