# Supplementary material for: The effects of exercise based on adherence to ACSM recommendations on pulmonary function and quality of life in adults with asthma: a systematic review and meta-analysis
Source: Front Physiol. 2025 May 15;16:1548382. doi: 10.3389/fphys.2025.1548382 (PMC12119264; doi:10.3389/fphys.2025.1548382)
Supplement: Supplementary file 9 [file DataSheet1.docx]

|  | Cochrane |
| --- | --- |
| #1 | (Asthma) OR (Occupational Asthma) OR (Exercise-Induced Cough-Variant Asthma) OR (Asthmas) OR (Asthma, Bronchial)  39044 |
| #2 | (Bronchial Asthma) OR (Asthmas, Occupational) OR (Occupational Asthma) OR (Occupational Asthmas) OR (Asthma, Exercise Induced)  8209 |
| #3 | (Exercise-Induced Asthmas) OR (Exercise-Induced Asthma) OR (Exercise Induced Asthma) OR (Bronchospasm, Exercise Induced) OR (Bronchospasm, Exercise-Induced)  1504 |
| #4 | (Exercise-Induced Bronchospasms) OR (Exercise-Induced Bronchospasm) OR (Exercise Induced Bronchospasm) OR (Asthma, Cough-Variant) OR (Cough Variant Asthma)  533 |
| #5 | #1 OR #2 OR #3 OR #4  39102 |
| #6 | (Exercise) OR (Circuit-Based Exercise) OR (Exercises) OR (Exercise, Physical) OR (Exercises, Physical)  153718 |
| #7 | (Physical Exercise) OR (Physical Exercises) OR (Physical Activity) OR (Activities, Physical) OR (Activity, Physical)  116550 |
| #8 | (Physical Activities) OR (Exercise, Aerobic) OR (Aerobic Exercise) OR (Aerobic Exercises) OR (Exercises, Aerobic)  100942 |
| #9 | (Exercise, Isometric) OR (Exercises, Isometric) OR (Isometric Exercises) OR (Isometric Exercise) OR (Acute Exercise)  20110 |
| #10 | (Acute Exercises) OR (Exercise, Acute) OR (Exercises, Acute) OR (Exercise Training) OR (Exercise Trainings)  67989 |
| #11 | (Training, Exercise) OR (Trainings, Exercise) OR (Circuit Based Exercise) OR (Circuit-Based Exercises) OR (Exercise, Circuit-Based)  58955 |
| #12 | (Exercises, Circuit-Based) OR (Circuit Training) OR (Training, Circuit)  1644 |
| #13 | #6 OR #7 OR #8 OR #9 OR #10 OR #11 OR #12  201968 |
| #14 | #5 AND #13 1417 |
